# Human Adipose-Derived Mesenchymal Stem/Stromal Cells Handling Protocols. Lipid Droplets and Proteins Double-Staining

**DOI:** 10.3389/fcell.2018.00033

**Published:** 2018-04-04

**Authors:** Aldana D. Gojanovich, María C. Gimenez, Diego Masone, Tania M. Rodriguez, Ricardo A. Dewey, Laura R. Delgui, Diego M. Bustos, Marina Uhart

**Affiliations:** ^1^Laboratorio de Integración de Señales Celulares, IHEM, Universidad Nacional de Cuyo, CONICET, Mendoza, Argentina; ^2^Facultad de de Ciencias Veterinarias y Ambientales, Universidad Juan Agustín Maza, Mendoza, Argentina; ^3^Facultad de Ingeniería, Universidad Nacional de Cuyo, Mendoza, Argentina; ^4^Laboratorio de Terapia génica y Células Madre, IIB-INTECH, Universidad Nacional de San Martín, CONICET, Chascomús, Argentina; ^5^Facultad de Ciencias Exactas y Naturales, Universidad Nacional de Cuyo, Mendoza, Argentina

**Keywords:** mesenchymal stem/stromal cells, adipose tissue, characterization, adipogenic differentiation, lipid droplets, fluorescence microscopy, staining, Oil Red O

## Abstract

Human Adipose-derived mesenchymal stem/stromal cells (hASCs) are of great interest because of their potential for therapeutic approaches. The method described here covers every single step necessary for hASCs isolation from subcutaneous abdominal adipose tissue, multicolor phenotyping by flow cytometry, and quantitative determination of adipogenic differentiation status by means of lipid droplets (LDs) accumulation, and Western blot analysis. Moreover, to simultaneously analyze both LDs accumulation and cellular proteins localization by fluorescence microscopy, we combined Oil Red O (ORO) staining with immunofluorescence detection. For LDs quantification we wrote a program for automatic ORO-stained digital image processing implemented in Octave, a freely available software package. Our method is based on the use of the traditional low cost neutral lipids dye ORO, which can be imaged both by bright-field and fluorescence microscopy. The utilization of ORO instead of other more expensive lipid-specific dyes, together with the fact that the whole method has been designed employing cost-effective culture reagents (standard culture medium and serum), makes it affordable for tight-budget research laboratories. These may be replaced, if necessary or desired, by defined xeno-free reagents for clinical research and applications.

## Introduction

The concept of white adipose tissue (WAT) has changed dramatically over the last decades. The identification of stable mesenchymal stem/stromal cells (MSCs) in WAT, called human Adipose-derived MSCs (hASCs), transformed WAT from a passive energy depot to a promising stem cells source. Compared to human Bone Marrow-derived MSCs (hBMSCs), hASCs are more easily accessible, and their isolation yield higher amount of stem/stromal cells, without ethical concerns (Zhu et al., 2008). These advantages, in addition to those shared with other stem/stromal cells, make the hASCs of high interest for cell therapies and tissue engineering (Strem et al., 2005). For hASCs isolation, once the WAT is harvested through a liposuction procedure (liquefied form) or abdominal dermolipectomy (solid form), it is subjected to mechanical disruption and washing. Then, the hASCs are detached from the surrounding tissue by enzymatic digestion. Although a number of articles described this process (Zuk et al., 2001; Gimble and Guilak, 2003; Lee et al., 2004; Dicker et al., 2005; Locke et al., 2009; Bourin et al., 2013; Ge et al., 2016; Kim et al., 2016), most lack detailed technical information, depicting images, molecular characterization or quantification of adipogenic differentiation. In addition, to resolve a protein extract from adipogenic-linage differentiated hASCs by SDS-PAGE, higher concentration of detergent in the lysis buffer is required, an ignored fact in most articles. Here, we present a comprehensive protocol for hASCs isolation, culture, molecular characterization, adipogenic differentiation, LDs and cellular proteins double-staining [with Oil Red O (ORO) and direct or indirect immunofluorescence (IF), respectively], image-based LDs quantification, and protein analysis by Western blot (WB). The key features of our protocol are:

- Compared to previously reported, this protocol represents the most comprehensive contribution, covering every step from isolation of hASCs to quantitative determination of their adipogenic potential.
- We employed low-cost culture reagents, constituting a cost-effective tool for tight-budget laboratories. These may be replaced by defined xeno-free reagents for clinical applications.
- We utilized the traditional low-cost neutral lipids dye ORO, suitable for imaging both by bright-field and fluorescence microscopy. Our classical isopropanol-dissolved ORO-staining protocol was optimized to avoid dye precipitation.
- We combined ORO-staining with IF technique to simultaneously analyze LDs accumulation and localization of cellular proteins. To our best knowledge, this combination has not been previously described on cultivated cells.
- We wrote a program to automatically quantify cellular LDs in both bright-field and fluorescence images. This program is supplied as supplementary information.

LDs are present in a wide range of cell types both in health and disease (Thiam et al., 2013). Their biogenesis and maturation are related to lipid metabolism, which is unbalanced in insulin resistance, obesity, and other lipid-related human disorders. LDs carrying triacylglycerol (TAG) and cholesteryl esters are emerging as dynamic cellular organelles generated in nearly every cell, bearing key roles in lipid and membrane homoeostasis. Abnormal LDs dynamics are associated with the pathophysiology of many metabolic disorders as obesity, diabetes, atherosclerosis, fatty liver, and even cancer (Hesse et al., 2013). Moreover, multiple intracellular pathogens, including viruses, bacteria, and parasites, specifically target host LDs during their life cycle (Roingeard and Melo, 2017). Our manuscript could help researchers interested in basic science of MSC, LDs, as well as those interested in clinical studies. Besides hASC isolation it includes:

- Characterization by multicolor flow cytometry by using the Human MSC Analysis Kit from BD, based on the phenotypic signature described by the International Society for Cellular Therapy (ISCT) (Bourin et al., 2013) for reproducibly phenotyping expanded MSCs.
- Differentiation of hASCs into the adipogenic lineage. To induce differentiation, the adipogenic drug cocktail composition and the induction time line have been optimized.
- ORO-IF double staining to analyze proteins of interest in ORO-stained samples. These two procedures have been applied previously on skeletal muscle (Koopman et al., 2001), but not in cultured cells, in which other, more expensive, lipid-specific dyes have been combined with IF (Melo et al., 2011).
- LDs quantification. An automatic procedure for ORO-stained cell digital image processing was created.

Undoubtedly, this report will be of great value for stem/stromal cell and adipogenesis research, as well as for the analysis of LDs in a wide range of cell types and conditions, especially in combination with proteins of interest by the double staining (ORO-IF) technique.

## Materials and equipment

### Reagents

#### Antibodies

- Actin: Rabbit anti-actin-FITC (Sigma-Aldrich, St. Louis, MO, United States, catalog no.: F3777) and Mouse anti-actin (BD Biosciences - Becton, Dickinson and Company, Franklin Lakes, NJ, United States, catalog no.: 612656).
- Adipose Differentiation-Related Protein: Rabbit anti-ADRP (Abcam, Cambridge, United Kingdom, catalog no.: ab52355)
- Early Endosome Antigen 1: Mouse anti-EEA1 (BD Biosciences, catalog no.: 610456)
- 130 kDa cis-Golgi Matrix protein: Mouse anti-GM130 (BD Biosciences, catalog no.: 610822)
- Bovine anti-mouse IgG (Santa Cruz Biotechnology, Santa Cruz, CA, United States, catalog no.: sc-2371)
- Donkey anti-mouse IgG and donkey anti-rabbit IgG Alexa Fluor 488 (Thermo Fisher Scientific, Waltham, MA, United States, catalog no.: 21202 and 21206)

#### Other reagents

- 0.2 μm PVDF blotting membrane (GE Healthcare Life science, Buckinghamshire, United Kingdom, catalog no.: 10600021)
- 0.22 and 0.45 μm nitrocellulose membrane (GE Healthcare Life science, catalog no.: RPN2020D)
- 2-mercaptoethanol (Sigma-Aldrich, catalog no.: M-6250)
- 3-isobutyl-1 methylxanthine IBMX (Sigma-Aldrich, catalog no.: I5879)
- Accutase® (BD Biosciences, catalog no.: 561527)
- Acrylamide (Sigma-Aldrich, catalog no.: A-3887)
- Ammonium Persulfate, APS (Bio-Rad, Hercules, CA, United States, catalog no.: 161-0700)
- BD Stemflow™ hMSC Analysis Kit (BD Biosciences, catalog no.: 562245)
- Bovine serum albumin, BSA (Roche, Basilea, Switzerland, catalog no.: 10735078001)
- Bromophenol Blue (Biopack, Zárate, Buenos Aires, Argentina, catalog no.: 2000962201)
- Calcium chloride, CaCl_2_ (Sigma-Aldrich, catalog no.: 499609)
- Calf Serum (Natocor, Villa Carlos Paz, Córdoba, Argentina)
- Collagenase D (Roche, catalog no.: 11088882001)
- Coomassie Brillant Blue G250 (Bio-Rad, catalog no.: 1610406)
- Dexamethasone (Sigma-Aldrich, catalog no.: 46165)
- Dimethyl sulfoxide, DMSO (Biopack, catalog no.: 2000992007)
- Dulbecco's Modified Eagle's Medium DMEM (Thermo Fisher Scientific, catalog no.:12800058)
- Ethanol (Anedra, Tigre, Buenos Aires, Argentina, catalog no.: AN00927025)
- Ethylenediaminetetraacetic acid, EDTA (Biopack, catalog no.: 2000964506)
- Fetal Bovine Serum, FBS, Biotechnology Grade, heat inactivated (Internegocios, Mercedes, Buenos Aires, Argentina)
- Glacial acetic acid (Biopack, catalog no.: 2000165308)
- Glycerin (Biopack, catalog no.: 2000162008)
- Glycine (Sigma-Aldrich, catalog no.: G-8898)
- Hoechst (Sigma-Aldrich, catalog no.: H3570)
- Hydrochloric acid, HCl (Dalton, Mendoza, Argentina, catalog no.: 0004801000)
- Insulin Humulin R, recombinant, human, 100 UI/mL, sterile suspension (Eli Lilly and Company, Indianapolis, IN, United States, catalog no.: HI-210)
- Isopropyl alcohol (Anedra, catalog no.: AN00620291)
- Magnesium chloride hexahydrate, MgCl_2_.6H_2_O (Biopack, catalog no.: 2000962005)
- Methanol (Anedra, catalog no.: AN00619725)
- Mowiol 4-88 (Sigma-Aldrich, catalog no.: 81381)
- N, N, N′, N′-tetramethylethylenediamine, TEMED (Thermo Fisher Scientific, catalog no.: 15524010)
- N, N′-Methylenebisacrylamide (Sigma-Aldrich, catalog no.: 146072)
- Non-faty dry milk
- Oil Red O (Biopack, catalog no.: 2000967402)
- Paraformaldehyde, PFA (Sigma-Aldrich, catalog no.: 441244)
- Phenylmethylsulfonyl fluoride, PMSF (Sigma-Aldrich, catalog no.: P7626)
- PierceTM BCA Protein Assay Kit (Thermo Fisher Scientific, catalog no.: 23227)
- Ponceau S (Biopack, catalog no.: 2000963003)
- Potasium chloride, KCl (Biopack, catalog no.: 2000163107)
- Potasium monobasic phosphate, KH_2_PO_4_ (Biopack, catalog no.: 2000963506)
- Rosiglitazone (Laboratories Beta, Buenos Aires, Argentina, cat. no.: 476863-2)
- Saponin (Biopack, catalog no.: 2000946003)
- Sodium bicarbonate NaHCO_3_ (Sigma-Aldrich, catalog no.: S-6015)
- Sodium chloride, NaCl (Biopack, catalog no.: 2000164608)
- Sodium dibasic phosphate, Na_2_HPO_4_ (Merck Millipore, Billerica, MA, United States, catalog no.: 106559)
- Sodium dodecyl sulfate, SDS (Genbiotech, Sophia Antipolis, Maritime Alps, France, catalog no.: RU2408)
- Sodium hydroxide, NaOH (Sigma-Aldrich, catalog no.: 221465)
- Sodium monobasic phosphate monohydrate, NaH_2_PO_4_.H_2_O (Sigma-Aldrich, St. Louis, MO, United States, catalog no.: S-3522)
- Sulfosalicylic acid (Sigma-Aldrich, catalog no.: S-3147)
- Super Signal® West Pico Chemiluminescent Substrate (Thermo Fisher Scientific, catalog no.: 34077)
- Triclhoracetic acid (Merck Millipore, catalog no.: 100807)
- Trizma base (Sigma-Aldrich, catalog no.: T1503)
- Trypsin 1:250 (Thermo Fisher Scientific, catalog no.: 27250018)
- Tween-20 (Sigma-Aldrich, catalog no.: P9416)

### Other materials

- 0.60 μm nylon mesh (Merck Millipore, catalog no.: NY6000010)
- 1.5 mL polypropylene conical microcentrifuge tubes (Extra Gene, Taichung, Taiwan, China, catalog no.: TUBE-170-C)
- 10 cm diameter tissue culture plate (Jet Biofil®, Guangzhou, Cantón, China, catalog no.: TCD010100)
- 15 mL polypropylene conical centrifuge tubes (Biologix, Jinan, Shandong, China, catalog no.: 10-0152)
- 50 mL polypropylene conical centrifuge tubes (Extra Gene, Taichung, Taiwan, China, catalog no.: P1013-50BF)
- Cover slips round 12 mm (Thermo Fisher Scientific, catalog no.: 50-143-822)
- Cryovials (Extra Gene, catalog no.: CR-2.0EB-S)
- Filter, autoclavable 3 mm wire mesh round filter, 3.5 mm diameter
- Flask, autoclavable glass flask with the same diameter as the 3 mm wire mesh round filter.
- Neubauer chamber (Marienfeld, Lauda-Königshofen, Alemania, catalog no: 0610039094)
- Petri dish (Nest, Rahway, NJ, United States, catalog no.: 752001)
- Plastic bag, sliding closure polipropilene bag, 20 × 20 cm
- Scissors, autoclavable operating scissors, Vantage-Miltex. Inc., Plainsboro, NJ, United States, catalog no.: V95-22)
- Serological pipettes, sterile, disposable (Jet Biofil®, catalog no.: GSP012010)
- Surgical blades, gamma radiation sterilized (Holden medical B.V., Lelystad, The Netherlands, no. 3.13)
- Surgical forceps, autoclavable (Roboz Surgical Instrument Co., Inc., Gaithersburg, MD, United States, catalog no.: RS-8100)
- Whatman cellulose filter paper, Ashless Quantitative Filter Paper, 20–25 μm, 9 cm; (Whatman plc-GE Healthcare catalog no.: 1441-090 41)
-No datasets were generated for this study.

### Recipes

***ATTENTION*** All solutions described are prepared with Milli-Q quality water (or similar).

*10 X PBS* (Stock solution, 200 mL)

Dissolve 16 g of NaCl, 0.4 g of KCl, 2.84 g of Na_2_HPO_4_, and 0.54 g of KH_2_PO_4_ in 200 mL of water. Sterilize by autoclave. To prepare *1 X PBS* solution dilute 1:10 with sterile water before use.

***REST*** This solution may be kept at RT, 4°C, or −20°C for long term storage.

*1 X PBSCM* (50 mL)

Add 50 μL of 1 M MgCl_2_ and 50 μL of 1 M CaCl_2_ in 50 mL of 1 X PBS.

***REST*** This solution may be kept at RT, 4°C, or −20°C for long term storage.

*2% Collagenase* (Stock solution, 15 mL)

Dissolve 0.3 g of collagenase in 15 mL of 1 X PBS and sterilize by filtration through a 0.22 μm nitrocellulose membrane.

***REST*** Single use aliquots of this solutions are kept at −20°C for long term storage.

*2.5% Trypsin* (Stock solution, 100 mL)

Dissolve 2.5 g of Trypsin and 0.85 g of NaCl in 100 mL of water, and sterilize by filtration through a 0.22 μm nitrocellulose membrane.

***REST*** 12 mL aliquots of this solution are stored at −20°C. These aliquots may be thawed and frozen several times.

*0.5% Trypsin/EDTA* (Stock solution, 10 mL)

Dissolve 0.5 g de Trypsin, 0.15 g of EDTA, and 0.085 g of NaCl in 10 mL of water, and sterilize by filtration through a 0.22 μm nitrocellulose membrane.

***REST*** 1 mL aliquots of this solution are kept at −20°C for long term storage.

*Adipogenic differentiation Induction Cocktail* (10 mL)

**Table:** 

**Component**	**Volume (μL)**	**Final concentration**
2.5 mM Dexamethasone	1	250 nM
20 mM Rosiglitazone	1	2 μM
50 mM IBMX	100	0.5 mM
4.17 mg/mL Insulin (ready to use sterile suspension, commercial)	24	10 μg/mL

*TIP* Prepare this solution just before each use.

***ATTENTION*** The volumes indicated in the table above correspond to the one used for a single 10 cm diameter culture dish. It is necessary to scale up or down to the volume necessary, according to the culture well, dish or flask (and the number of them) to be used.

*2.5 mM Dexamethasone* (Stock solution, 1.25 mL)

Dissolve 12 mg of Dexamethasone in 1.25 mL of absolute ethanol, and sterilize by filtration through a 0.22 μm nitrocellulose membrane.

***REST*** 40 μL aliquots of this solution are stored at −20°C for up to 2 years.

*20 mM Rosiglitazone* (Stock solution, 840 μL)

Dissolve 8 mg of Rosiglitazone in 840 μL of DMSO, centrifuge 10 min at 25°C, and sterilize by filtration through a 0.22 μm nitrocellulose membrane.

***REST*** 40 μL aliquots of this solution are stored at −80°C for up to 2 months.

*50 mM 3-isobutyl-1 methylxanthine* (IBMX, Stock solution, 500 μL)

Dissolve 5.75 mg of 3-isobutyl-1 methylxanthine in 450 μL of water with 50 μL of NaOH, and sterilize by filtration through a 0.22 μm nitrocellulose membrane.

***REST*** 40 μL aliquots of this solution are stored at −20°C for up to 3 months. These aliquots may be thawed and frozen several times.

*4% PFA solution, pH 7.2–7.4* (50 mL)

Dissolve 2 g of PFA in 10 mL of water at 80°C, add approximately 5 mL of 16 N NaOH (drop by drop) while stirring until the PAF is dissolved. Then add 20 mL of 1% NaH_2_PO_4_ solution and 20 mL of 1.6% Na_2_HPO_4_ solution. Once the solution is cold adjust the pH with 3 N HCl.

***REST*** 4 mL single use aliquots of this solution are kept at −20°C for long term storage.

***ATTENTION*** Once thawed, these aliquots should not be frozen again, because of the risk of crystallization.

*0.35% Oil Red O* (Stock solution, 10 mL)

Dissolve 0.35 g of Oil Red O in 10 mL of isopropyl alcohol. Filter twice, first through a 0.45 μm, and then through a 0.22 μm nitrocellulose membrane. To prepare the *working solution*, which should always be done just before use, dissolve 6 parts of ORO stock solution in 4 parts of water, and filter through a 0.22 μm nitrocellulose membrane.

***REST*** ORO stock solution can be stored for up to 2 months at RT and protected from light.

***ATTENTION*** Always pay special attention to the presence of precipitates before using this solution. Discard if precipitates are found.

*2.5% Saponin/10% BSA* (Stock solution, 10 mL)

Dissolve 0.25 g of saponin and 1 g of BSA in 10 mL of water.

***REST*** 1 mL aliquots of Saponin/BSA solution are kept at −20°C for long term storage.

*13.3% Mowiol* (18 mL)

Dissolve 2.4 g of Mowiol 4–88 in 6 g of Glycerol. Add 6 mL of water and leave for 2 h at RT. Add 12 mL of 0.2 M Tris-HCl (pH 8.5) and incubate at 50°C for 10 min with occasional mixing. After the Mowiol dissolves, clarify by centrifugation at 5,000 × *g* for 15 min.

***REST*** 1 mL aliquots of this solution are kept at −20°C for long term storage. These aliquots are stable at room temperature or 4°C for several months after thawing.

*Cell Lysis Buffer* (20 mL)

Dissolve 0.14 g of Tris (Trizma base) and 0.4 g of SDS in 15 mL of water. Once dissolved, adjust the pH to 6.8 pH with 3 N HCl. Add distilled water up to 20 mL. Just before use add 10 M PMSF.

***REST*** This solution is stable at RT for several months.

*5 X Protein sample buffer* (Stock solution, 10 mL)

Dissolve 10 mg of bromophenol blue, 1 g of SDS, 5 mL of Glycerol, 2.5 mL of 1 M Tris-HCl (pH 6.8), and 0.5 mL 2-mercaptoethanol (see below) in water.

***REST*** 1 mL aliquots of this solution are kept at −20°C for long term storage without the addition of 2-mercaptoethanol. After thawing, add the corresponding volume of this reagent. These aliquots are stable at RT or 4°C for several months after thawing.

*0.25% Coomassie Brillant Blue* (300 mL)

Dissolve 0.75 g of Coomassie Brillant Blue G-250 in 270 mL of ethanol:water (1:1 v/v) and 30 mL of glacial acetic acid. Filter the solution through a Whatman filter paper to remove particulate matter.

***REST*** This solution is stable at RT for several months. It may be reused many times.

*2% Ponceau S* (100 mL)

Dissolve 2 g of Ponceau S, 30 g of triclhoracetic acid, and 30 g of sulfosalicylic acid in 100 mL of water.

***REST*** This solution is stable at RT for several months. It may be reused many times.

*12% Acrylamide Resolving gel* (7.5 mL)

**Table:** 

**Component**	**Volume (mL)**	**Final concentration**
Glycerol	1	13.40%
1.5 M Tris-HCl, pH 8.8	1.9	0.38 M
30% Acrylamide/0.8% Bisacrylamide	3	12%/0.30%
20% SDS	0.037	0.10%
TEMED	0.003	0.04%
10% APS	0.037	0.05%
H_2_O	1.45	–

*7.4% Acrylamide Stacking gel* (2.5 mL)

**Table:** 

**Component**	**Volume (mL)**	**Final concentration**
1 M Tris-HCl pH 6.8	0.320	0.13 M
30% Acrylamide/0.8% Bisacrylamide	0.620	7.4%/0.2%
20% SDS	0.0125	0.10%
TEMED	0.0025	0.10%
10% APS	0.025	0.10%
H2O	1.5	–

*10 X Laemmli Running Buffer* (Tris-glycine, Stock solution, 500 mL)

Dissolve 15.2 g of Tris (Trizma base), 72.1 g of Glycine, and 5 g of SDS in 500 mL of water. To prepare 300 mL of the 1 X running buffer dilute 1:10 with water.

***REST*** This solution is stable at RT for several weeks.

***ATTENTION*** Do not adjust the pH, as the addition of electrolytes would cause the current to increase too much. Discard if this solution turns yellowish, which is due to Glycine oxidation.

*1 X Towbin Protein Transfer Buffer, pH 8.3* (1 L)

Dissolve 3.03 g of Tris (Trizma base), 14.4 g of Glycine, and 1 g of SDS in 800 mL of water. Add methanol up to 1 L.

***REST*** This solution is stable at RT for several weeks.

***ATTENTION*** Do not adjust the pH as the addition of electrolytes would cause the current to increase too much.

*0.1% PBST* (Stock solution, 200 mL)

Add 200 μL Tween-20 to 200 mL of 1 X PBS. To prepare 200 mL of the 0.01% PBST solution dilute 1:10 with 1 X PBS before use.

***REST*** This solution is stable at RT for several months.

*5% Western blot blocking solution* (10 mL)

Dissolve 0.5 g of non-fat dry milk in 10 mL of 1 X PBS or 0.01% PBST.

***REST*** This solution is ideally prepared just before, and may be stored at 4°C for a few days. Addition of preservatives (as 0.02% Sodium azide) may prolong its shelf life.

### Equipment

- ZEISS Primo Vert Inverted Microscope (Zeiss; Oberkochen, Baden-Württemberg, Germany)
- Class II Biological Safety Cabinet (Biobase; Zhangqiu, Shandong, China)
- Sanyo MCO-19AIC CO_2_ cell incubator (Sanyo; Moriguchi, Osaka, Japan)
- Innova 4000 Incubator Shaker (New Brunswick Scientific, Enfield, CT, USA)
- Jouan B4i Multifunction Centrifuge (Thermo Fisher Scientific, Waltham, MA, USA)
- Accuri^TM^ C6 Cytometer (BD Biosciences)
- Eclipse TE2000 Inverted Microscope (Nikon, Shinagawa, Tokyo, JPN)
- ImageQuant™ LAS 4000 (GE Healthcare Life Sciences, Chicago, IL, USA)
- Reusable bottle-top filters (Note: Convenient, disposable bottle-top filters are available from a number of manufacturers. Reusable bottle-top filters provide a more cost-effective solution for laboratories on a more restricted budget).

## Stepwise procedures

### Isolation of human adipose-derived mesenchymal stem/stromal cells (hASCs)

**Figure 1:**
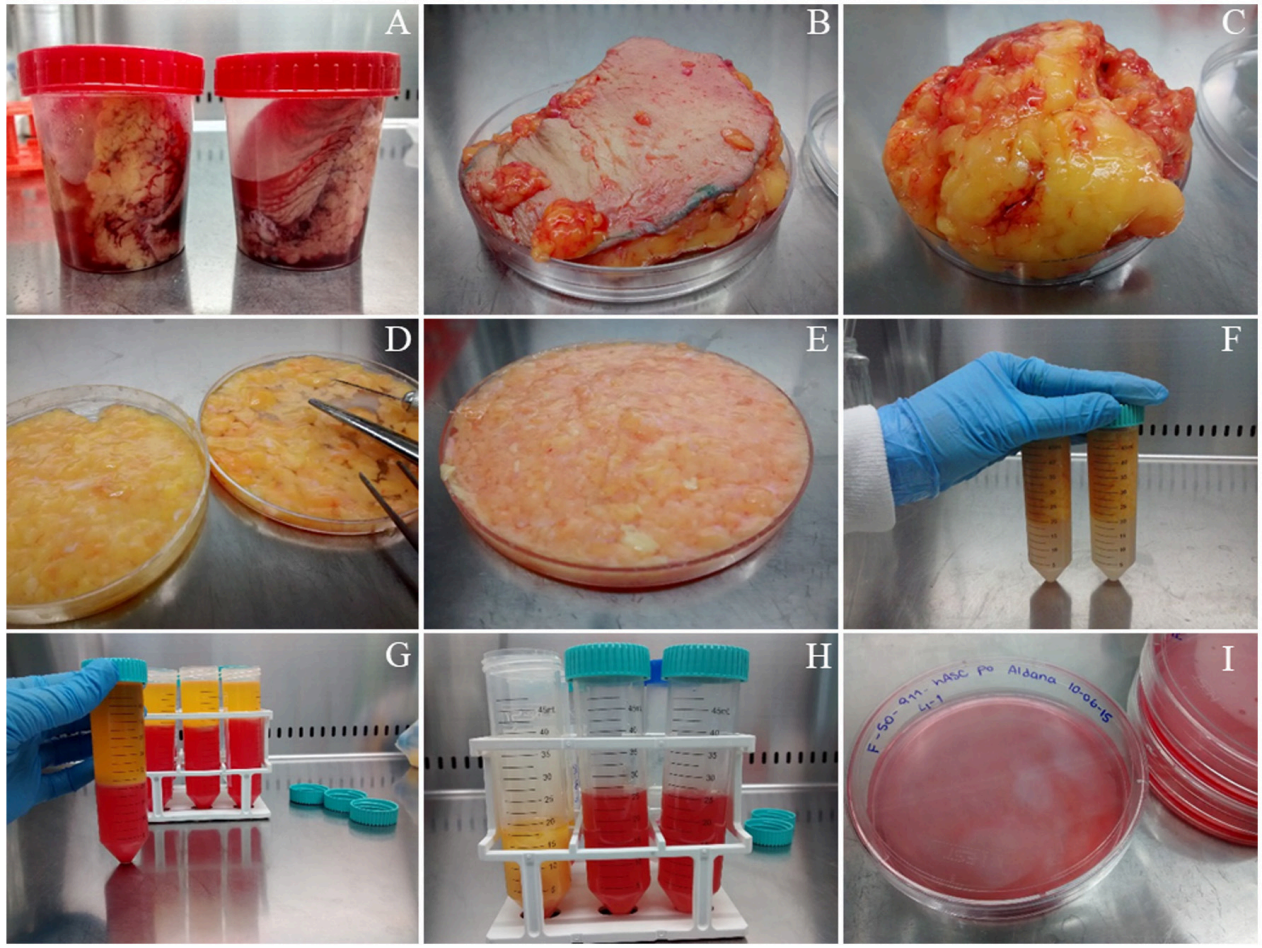
Isolation of mesenchymal stem/stromal cells from human solid adipose tissue**. (A)** solid white adipose tissue samples are received in 120 mL disposable sterile plastic containers, **(B)** the samples are placed on a Petri dish before starting their dissection, **(C)** fat is separated from skin (which is discarded), **(D)** fat tissue is cut with scissors, **(E)** fat is cut further with surgical blades, **(F)** fat is rinsed with PBS buffer in polypropylene conical tubes, **(G)** obtaining of three phases after filtration and centrifugation, **(H)** separation of the stromal vascular fraction (SVF) from the other two phases, **(I)** seeding the washed SVF in 10 cm diameter plate with supplemented DMEM.

***ATTENTION*** As a source of hASCs, we routinely use subcutaneous white adipose tissue (either solid or lipoaspirates) obtained from healthy donors undergoing esthetic surgical procedures. A picture of the way we receive solid tissue samples can be found in Figure 1A. This protocol was developed in accordance with the recommendations of the *Universidad Nacional de Cuyo* Bioethics guidelines, *Universidad Nacional de Cuyo* Bioethics Committee. The protocol was approved by the *Universidad Nacional de Cuyo* Bioethics Committee. All subjects gave written informed consent in accordance with the Declaration of Helsinki.

***REST*** The donated tissue may be stored at 4°C until processing. We routinely do it the day after, but have had good results from solid tissue stored for up to 3 days. In lipoaspirates the hASCs are not preserved so well, thus, processing should be done as soon as possible.

#### Initial notes

- All human-originated biological material must be considered as pathological, and as such, extreme care and adequate personal protective equipment should be used during manipulation. An appropriated protocol for the disposal of human material should be followed according to your Institution's pathological wastes.
- All solutions and equipment coming into contact with cells must be sterile, and an aseptic technique should be used accordingly, in a Class II biological safety cabinet.
- All centrifugation steps are performed at room temperature (RT) unless other temperature is specified.
- We routinely employ 10 cm diameter culture plates, although 75 cm^2^ cell culture flasks may be used as well.
- All cellular cultures are performed in a humidified 37°C, 5% CO_2_ incubator. We routinely utilize high glucose supplemented Dulbecco's Modified Eagle Medium (DMEM), which is composed by: high glucose DMEM, 10% Fetal Bovine Serum (FBS, 1 h inactivated at 60°C), 100 U/mL Penicillin-100 μg/mL Streptomycin.

1. *If the starting material is solid adipose tissue*, place it on a sterile Petri dish employing sterile forceps, see Figure 1B. Leave the dish lid aside to support the surgical instruments. Remove the skin, Figure 1C, and cut small pieces of adipose tissue (yellow colored, Figures 1D,E from the same panel) while removing blood vessels and connective tissue (whitish-pink colored) with sterile scissors. This step takes ~30 min/100 g of fat tissue.
2. Wash four times with Phosphate Buffer Saline (PBS: 0.13 mM NaCl, 2.70 mM KCl, 10 mM Na_2_HPO_4_, 2 mM KH_2_PO_4_, pH 7.4) placing the fat tissue pieces in a 50 mL polypropylene conical tube, close hermetically and shake. Discard the PBS using a 10 mL serological pipette. Repeat the wash three more times. When the PBS looks clean, without blood, Figure 1F, place the washed fat again on the Petri dish to continue the dissection. We usually obtain clean material after four washes, but more washes could be performed if needed. After the washing step, small blood vessels become more visible, which facilitates their removal with scissors. Chop further the adipose tissue pieces with the aid of a surgical scalpel and clamps until obtaining a soft consistency. This step takes ~30 min. and yield 60 mL of processed fat/100 g of fat tissue.
3. *If the starting material is lipoaspirate (fat from a liposuction procedure), the protocol starts at this current step, or continue the protocol started within the previous steps from solid tissue*. Wash with PBS as explained in step 2. For each wash add PBS, shake and centrifuge the tube at 200 × *g* in a swinging bucket rotor for 3 min. Remove the PBS with a 10 mL serological pipette.
  ***TIP*** As the PBS will be below the adipose tissue, and to avoid blocking the pipette, expel air as you go down vertically through the adipose phase with the pipette. Wash until the PBS looks clean (without blood), usually twice if the starting material was solid fat, or up to 5 washes if you started from lipoaspirate. These washes take ~45 min for 60 mL of fat divided in two conical tubes.
4. Transfer the fat to a fresh 50 mL conical tube without exceeding 30 mL of fat/tube. If you have more than 30 mL of fat, divide it into two tubes. Add trypsin and collagenase to final concentrations of 0.25 and 0.1%, respectively, to digest the remnant tissue pieces. Incubate 30–40 min at 37°C with gentle shaking in horizontal position. Alternatively, decreasing collagenase concentration to 0.05% with 1 h incubation time gives also enough tissue digestion.
  ***TIP*** It may be required to adjust optimal enzyme concentrations. Collagenase lots may vary in activity, and too much activity may kill the cells. However, too low activity would not digest enough the tissue for an adequate stem/stromal cells release. The incubation time may also depend on the enzyme lots and should be adjusted as necessary.
  ***ATTENTION*** Place the tubes tightly closed inside a hermetically closed plastic bag, and put the bag inside a plastic container for biological safety.
5. Add enough supplemented DMEM to double the initial fat volume, or if such volume is more than 25 mL, add supplemented DMEM up to 50 mL. This step inactivates the digestive enzymes.
6. Filter through a 3 mm wire mesh while mixing (above the filter) with a tip to prevent the connective tissue to clog the filter. Collect the liquid phase (supplemented DMEM + dissociated cells) in a wide opening flask with the same diameter as the filter, which has been firmly secured to the flask opening with paper tape and autoclaved. This step takes ~20 min for 60 mL of digested tissue.
7. Transfer the liquid filtrate to a 50 mL conical tube and centrifuge in a swinging bucket rotor for 10 min at 400 × *g* to obtain three phases that may be clearly visualized in Figure 1G: (i) the upper yellowish liquid layer composed of oil released from broken adipocytes; (ii) the middle white compact layer which contains unbroken adipocytes and pre-adipocytes; and (iii) a reddish aqueous phase (the color is due to the phenol red indicator included in DMEM medium) with a cellular pellet called stromal vascular fraction (SVF), which includes an heterogeneous population with mature blood, stem, stromal, and progenitor cells. Transfer the lower phase, including the SVF to a new Falcon tube, Figure 1H.
8. Centrifuge in a swinging bucket rotor for 10 min at 400 × *g* to re-obtain the SVF.
9. To wash the SVF, first, discard the supernatant, then, re-suspend the pellet (SVF) in 15 mL of DMEM and finally centrifuge again, as described in step 8.
  ***TIP*** This pellet is extremely labile; thus care should be taken when aspirating the media from above to avoid aspirating it.
10. Suspend the SVF in 10 mL of supplemented DMEM and transfer it to a 10 cm diameter tissue culture plate, Figure 1I. Culture the cells in a humidified 37°C, 5% CO_2_ incubator. Steps from 7 to 10 take ~45 min.
11. After 24 h, take the plate out of the incubator taking care of moving it as little as possible. It is expected to find turbidity in the culture medium due to the presence of a large amount of floating and/or decanted red blood cells. Employing a phase-contrast inverted microscope, look for adherent cells with fibroblastoid morphology attached to the bottom of the plate. These often appear surrounded by a small circle deprived of red blood cells (too much movement of the plate would disassemble this pattern, which is useful for rapidly detecting the adherent cells). Gently, wash 3 times with PBS and add fresh supplemented DMEM. These washes remove specifically red blood cells keeping the adherent cells, which correspond mainly to mesenchymal stem and/or stromal cells, in this case, hASCs. This firstly obtained hASCs population is considered passage “0.”
  ***TIP*** Although the cells are firmly attached to the plastic surface, it is always recommended to work gently, especially when adding media or PBS to the cell culture. These should be warmed at 37°C before being added to the cells. The addition should be done carefully, through the culture plate walls, and not directly over the cells, as hASCs are susceptible to stressful manipulation.
12. Wash and add fresh supplemented DMEM every day until red blood cells are not further detected.
13. Continue feeding the hASCs with fresh supplemented DMEM every 2 or 3 days until the monolayer reaches ~90% confluency.
  ***TIP*** Never allow the hASCs to reach 100% confluency as this may cause spontaneous differentiation and lose of the multipotency potential.

### hASCs expansion

14. Discard the medium and wash the cell monolayer twice, each time with ~5 mL 37°C pre-warmed sterile PBS.
15. Detach the hASCs with warm 0.05% trypsin/EDTA solution (500 μL is enough for a 10 cm diameter plate). It is important to minimize the exposure to trypsin/EDTA as the cells will die. Place the plate with trypsin into the cell incubator for 30 s, the cells will “round up” when detached.
  ***TIP*** Closely monitor the cells under the inverted microscope to identify when most of the cells are detached. It may be necessary to help mechanically, through gently tapping the plate by its side. All this procedure should take <2 min.
16. Add 6 volumes (3 mL) of supplemented DMEM to the trypsin reaction, and collect the cells employing a sterile serological pipette. Transfer the cells to a 15 mL conical tube.
17. Pellet the cells by centrifugation in a swinging bucket rotor at 400 × *g* during 5 min.
18. Discard the supernatant (trypsin-containing medium) and replace it with 1 mL of fresh supplemented DMEM.
19. Employ an aliquot (10 μL) to count the cells in a Neubauer chamber. Apply the following formula: cells/mL = (number of cells counted/number of quadrants) ^*^ 10^4^. Dilute as needed to seed flasks, plates, or wells with 0.7–1 × 10^4^ cells/cm^2^.

### hASCs characterization by flow citometry

**Figure 2:**
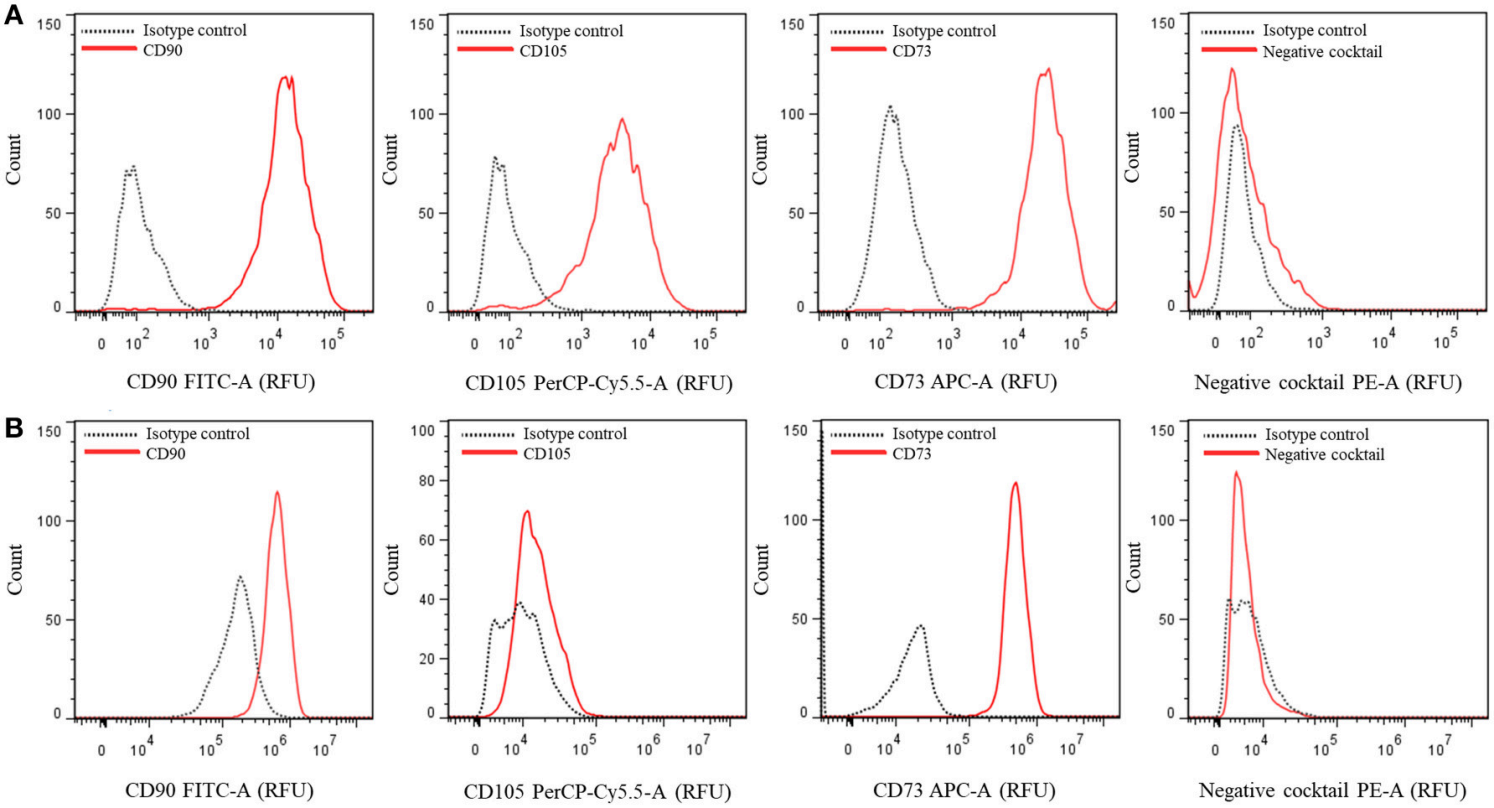
Multicolor phenotyping of hASCs by flow citometry. Histograms represent the number of events (count) vs. the relative fluorescence units (FRU) for each indicated fluorophore conjugated-antibody or cocktail. **(A)** Representative graphs of a population with most of the cells positive for CD90, CD105, and CD73. **(B)** Representative graphs of a different population of cells which did not express CD105.

20. To characterize hASCs, we routinely employ the “BD Stemflow™ hMSC Analysis Kit” for reproducible multicolor phenotyping of mesenchymal stem cell expansions (BD Biosciences). This kit includes a mixture of three antibodies (conjugated with different fluorophores) that recognize hMSCs positive surface markers: CD90-FITC, CD105-PerCP-Cy^TM^ 5.5 and CD73-APC (positive cocktail), and a mixture of 5 single fluorophore (PE)-labeled antibodies that recognize surface markers of other cell types different from hMSCs: CD45, CD34, CD11b, CD19, and HLA-DR (negative cocktail). For compensation, CD90-FITC, CD44-PE, CD105-PerCP-Cy^TM^ 5.5, and CD73-APC are used. The kit also includes the corresponding isotype controls.
21. Grow the hASCs to 90% confluency in a 10 cm diameter culture plate under standard conditions (supplemented DMEM, 37°C and 5% CO_2_).
22. Wash with PBS buffer and detach the cells by addition of 500 μL of Accutase® (BD Biosciences). This is a natural enzyme mixture with proteolytic and collagenolytic enzyme activity, which preserves most epitopes for subsequent flow cytometry analysis. Incubate for 10 s at 37°C.
23. Inhibit the enzymatic reaction by adding 3 mL of supplemented DMEM, which neutralizes the enzymes activity. Collect the cells by gentle pipetting.
24. Transfer the cells into a 15 mL conical tube and centrifuge at 200 × *g* for 5 min at RT.
25. Discard the supernatant and re-suspend the cell pellet with 600 μL of a solution made of 1% v/v calf serum in PBS (Wash and Label Solution, WLS). Filter through a 0.60 μm nylon mesh.
26. Count the cells in a Neubauer chamber, and place 50,000 cells in each cytometer tube together with the corresponding antibodies (0.5–2 μL/tube with cells), following the instructions from the manufacturer.
27. Incubate the tubes on ice, in the dark, for 30 min.
28. Washing step: add 200 μL of WLS and centrifuge at 200 × *g* for 5 min. Repeat this step a second time and after the second wash, remove the supernatant.
29. Re-suspend the pellet in 150 μL of PBS.
30. Analyze and quantify the samples by using the FlowJo software version 7.6 in an Accuri^TM^ C6 cytometer (BD Biosciences, USA) or equivalent, Figure 2.

### Cryopreservation

31. Following expansion, it is recommended to cryopreserve hASCs at passage 2.
32. When the cell density reaches ~0.8–1 × 10^4^ cells/cm^2^ (which corresponds to a coverage of 80–95% of the plate surface with attached cells), proceed as described in steps 14–17 to wash and detach the cells.
33. After centrifugation, discard the trypsin-containing medium and replace it with freezing medium (10% FBS, 10% DMSO, DMEM) at a volume of 1 mL/10 cm diameter culture plate employed for producing the pellet. This will result in a freezing density of ~5–8 × 10^5^ cells/mL.
34. Employ a 5 mL serological pipette to suspend the cells, then place 1 mL aliquots of cells into cryovials and place them immediately into a controlled freezing system as the one explained in the next step.
35. hASCs require a controlled freezing rate of −1°C/min., otherwise the mortality reaches almost 100%. Many systems are currently available to control the freezing rate. We use an alcohol-based cell freezing container, which requires fresh supply of 100% isopropyl alcohol, to maintain an approximate −1°C/min freezing rate. Place the cryovials into the container with isopropyl alcohol at RT, and the container in an −80°C freezer for at least 16 h.
36. The next day (or within the week), transfer all cryovials into a liquid nitrogen tank, for long-term storage.
37. To culture the frozen cells, transfer the cryovials from the liquid nitrogen tank to a 37°C water bath. Thaw until a small piece of ice is visible inside the cryovial, and pour it in 5 or 6 volumes of 37°C pre-warmed supplemented DMEM contained in a plastic conical tube. Gently move the closed tube to wash out the DMSO from the cells.
38. Pellet the cells by centrifugation in a swinging bucket rotor at 400 × *g* during 5 min.
39. Discard the supernatant (DMSO-containing medium) and replace it with 10 mL of 37°C pre-warmed supplemented DMEM.
40. Pipette gently and transfer to a 10 cm diameter-culture plate or T-75 flask.
  ***TIP*** Cells will grow to 100% confluency in about 3–7 days after trypsinization or cryopreservation if the starting confluency is above 30%. Seeding densities lower than 30% usually result in the change of the typical fibroblast morphology to widen, expanded, and highly branched, and in a decrease of proliferation capacity. Proliferation of hASCs also dramatically decreases by passage 10, when the shape gradually changes again from fibroblastic to expanded and branched. Experiments should be done ideally with hASCs at passages 3–6.

### hASCs adipogenic differentiation

**Figure 3:**
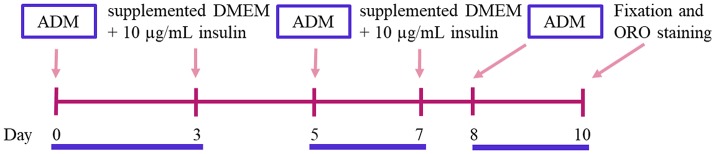
Adipogenic differentiation induction time line. ADM, Adipogenic Differentiation Medium; ORO, Oil Red O.

**Figure 4:**
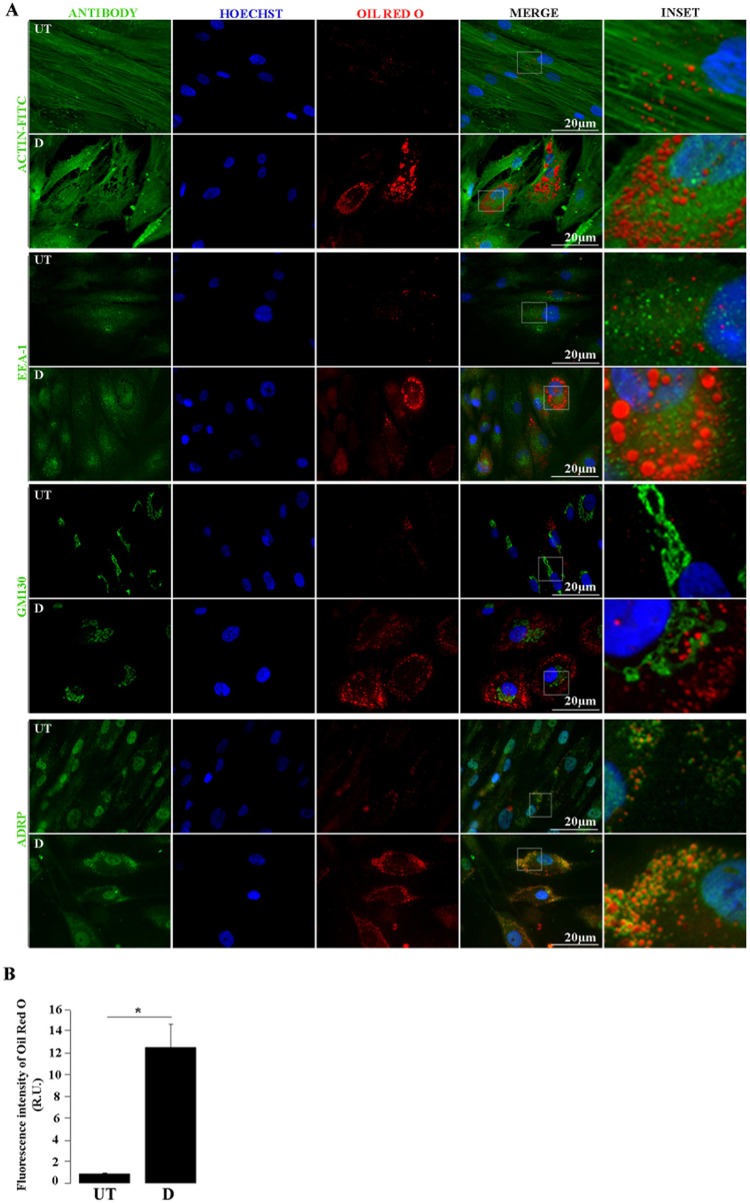
Lipid droplets and cellular proteins double staining. **(A)** by Untreated (UT) and differentiated (D) hASCs were Oil Red O (ORO) and immunostained, and analyzed by fluorescence microscopy. Green signal corresponds to the indicated proteins detected by immunofluorescence (Actin-FITC) or indirect immunofluorescence (EEA-1, GM 130, ADRP); blue signal corresponds to Hoechst-stained nuclei; red signal corresponds to ORO-stained LDs. **(B)** Automated quantification of ORO fluorescence intensity relative units (R.U.) in UT and D hASCs. Bars represent mean values (+/– standard deviation) from 14 ORO-stained images prepared as in A and normalized to the number of nuclei/image. The asterisk indicates significant differences between samples (Student's test; *p*-value < 0.01). Data were similar in two other experiments.

41. Place a sterile round cover slip (10–12 mm diameter) in each well of a 24-well plate to be used.
42. Seed 45,000 hASCs/well. After 24–36 h, the cells should reach 80–90% confluency.
43. Replace the culture medium by the induction one, consisting of supplemented DMEM with 250 nM dexamethasone, 0.5 mM 3-isobutyl-1 methylxanthine (IBMX), 2 μM rosiglitazone and 10 μg/mL insulin. This will be considered day 0 (Figure 3).
  ***TIP*** Stock solutions are prepared, stored and used as detailed below, under the RECIPES section.
44. Incubate the cells with induction medium during 3 days, followed by 2 days in supplemented DMEM with 10 μg/mL insulin. Untreated (control) cells are grown in 5% v/v FBS supplemented DMEM, which is changed using the same schedule applied to the treated cells until the end of the experiment (Figure 3).
45. A second induction step with the adipogenic cocktail is left for 2 additional days, followed by 1 day incubation in supplemented DMEM with 10 μg/mL insulin (Figure 3).
46. Finally, a third and last induction is left for 2 additional days (Figure 3).
47. Lipid droplets (LDs), which look like bright refractive round structures on a phase-contrast inverted microscope, become visible at day 5 after induction. The LDs grow in size and number as the adipogenic differentiation progresses.
  3.6. Fixation and Oil Red O staining
48. Prepare 0.35% w/v ORO (Biopack, Argentina) stock solution in isopropanol and incubate overnight at RT as detailed in the RECIPES section. Filter the stock solution twice, first through a 0.45 μm and then through a 0.22 μm nitrocellulose membrane.
  ***ATTENTION*** This step is CRITICAL, as the presence of precipitates would prevent visualization and accurate quantification of ORO-stained LDs. Before using this solution it is again CRITICAL to pay attention to the formation of precipitates. If this occurs discard this stock solution to avoid plugging the nitrocellulose filter and start this step (number 45) again.
49. Wash the cells three times with PBS containing 1 mM CaCl_2_ and 1 mM MgCl_2_.
50. Fix the cells with 4% w/v paraformaldehyde (PFA, 400 μL/well of a 24-well plate) for 20 min at RT.
51. Wash three times with PBS.
52. Dilute the ORO stock solution in ultrapure water (6:4 ratio), incubate 20 min at RT and filter through a 0.22 μm nitrocellulose membrane.
53. Add 300 μL/well (of a 24-well plate) of ORO working solution to the fixed cells and incubate for 2 h at RT with gentle shaking and protected from light.
54. Exhaustively rinse the monolayer with ultrapure water until it looks uncolored.
55. Mount the cover slips in an appropriate mounting medium (e.g., Mowiol, 5 μL/12 mm diameter cover slip) for bright field and fluorescence microscopy analysis, or proceed with step 53 to perform an indirect immunofluorescence (IFI) staining.
  3.7. Immunofluorescence (IF) of ORO-stained samples.
56. After ORO-staining, incubate the cover slips with 0.05% w/v saponin in PBS containing 0.2% w/v bovine albumin (BSA), for 20 min.
  ***TIP*** This step includes permeabilization (due to saponin) and blocking (due to albumin). The former allows the antibodies to cross the hASCs membranes, and the later avoids unspecific binding.
57. Wash three times with PBS, incubating each time for 5 min with gentle shaking.
58. Incubate the monolayer with the appropriate dilution of primary antibodies in PBS overnight (ON) at 4°C. The primary antibodies we used were detailed on the REAGENTS section.
59. Wash with PBS as in step 54, and then incubate with the appropriate dilution of fluorescently labeled secondary antibodies in PBS at RT for 1.5 h. The secondary antibodies used in our assay were detailed on the REAGENTS section.
  ***TIP*** If the primary antibody is conjugated to a fluorophore (as anti-actin-FITC in our assay), this step is unnecessary.
60. Wash with PBS as in step 51. Mount with Mowiol (including 10 μg/μL Hoechst stain or similar to stain the nucleus).
61. Observe and take images by fluorescence microscopy (Figure 4A) or laser scanning confocal microscopy. The combination of filters used in our example was as follows: Hoeschst, excitation band-pass filter (BPF) 330/385, dichroic mirror (DM) 445/585, emission BPF 450/485; FITC or Alexa Fluor-488, excitation band-pass filter (BPF) 460/490, dichroic mirror (DM) 505/520, emission BPF 520/545; Red Oil O, excitation band-pass filter (BPF) 520/550, dichroic mirror (DM) 560/580, emission BPF 590/650.

### Images quantification

***TIP*** The following steps describe the procedure to automatically quantify ORO-stained cell images using the freely available software package Octave (Eaton et al., 2015). Basically, we quantified the number of red-colored pixels in fluorescence or bright field images and the number of cells from DAPI or Hoechst-stained nuclei.

62. Copy and paste all images to analyze in a new folder.
63. Use Octave's image processing functions described by Eaton et al. (2015) to process images (following steps listed in 62 b-g), or copy and paste our Octave program “OilRed.m” (freely available upon request) into the same folder and open Octave package.
64. Open OilRed.m and run the file. The program will determine the number of red pixels in all images and will generate a file containing all image names with their respective pixels quantification.

While being executed, the program OilRed.m automatically performs the following steps:

(a) In Octave, it opens the first image (in either.jpg or.tiff format).
(b) Adjusts colors using the function “imadjust” to intensify red.
(c) Defines colors' upper and lower limits.
(d) Quantifies pixels falling in these intervals (defined in c) for the current image.
(e) Saves pixels' quantification and image files' names.
(f) The program automatically repeats steps from (a) to (f) with every image until all of them have been processed.
(g) Generates a file “Table 1.txt”, containing all image files' names with their respective pixels quantification.

65. To quantify the number of cells within each image, copy and paste all images to analyze in a new folder.
66. Use Octave's image processing functions described by Eaton et al. (2015) to process images (following steps listed in 65 b–h), or copy and paste our Octave program “CellCount.m” (freely available upon request) into the same folder and open Octave package.
67. Open and run CellCount.m file, the program will count cells within the images and will generate a file containing all image files' names with their respective cell number quantification.
68. While being executed, the program CellCount.m performs the following automatic steps:

(a) In Octave, it opens the first image (in either.jpg or.tiff format).
(b) Adjusts colors using function “imadjust” to intensify the color of interest.
(c) Converts image to gray scale (250 tones) using the function “rgb2gray.”
(d) Converts image to black and white (2 tones) using the function “im2bw.”
(e) Fills small holes in the black and white image using the function “imfill.”
(f) Quantifies cells using the function “bwlabel.”
(g) Repeats from (a) to (f) with the next image until all of them have been processed.
(h) Generates a file “Table 2.txt”, containing all image files' names with their respective cell quantification, which may be used to normalize the values obtained in step 64(g), statistically analyzed and graphed as in Figure 4B.

### Western blot analysis of adipogenic differentiated hASCs

**Figure 5:**
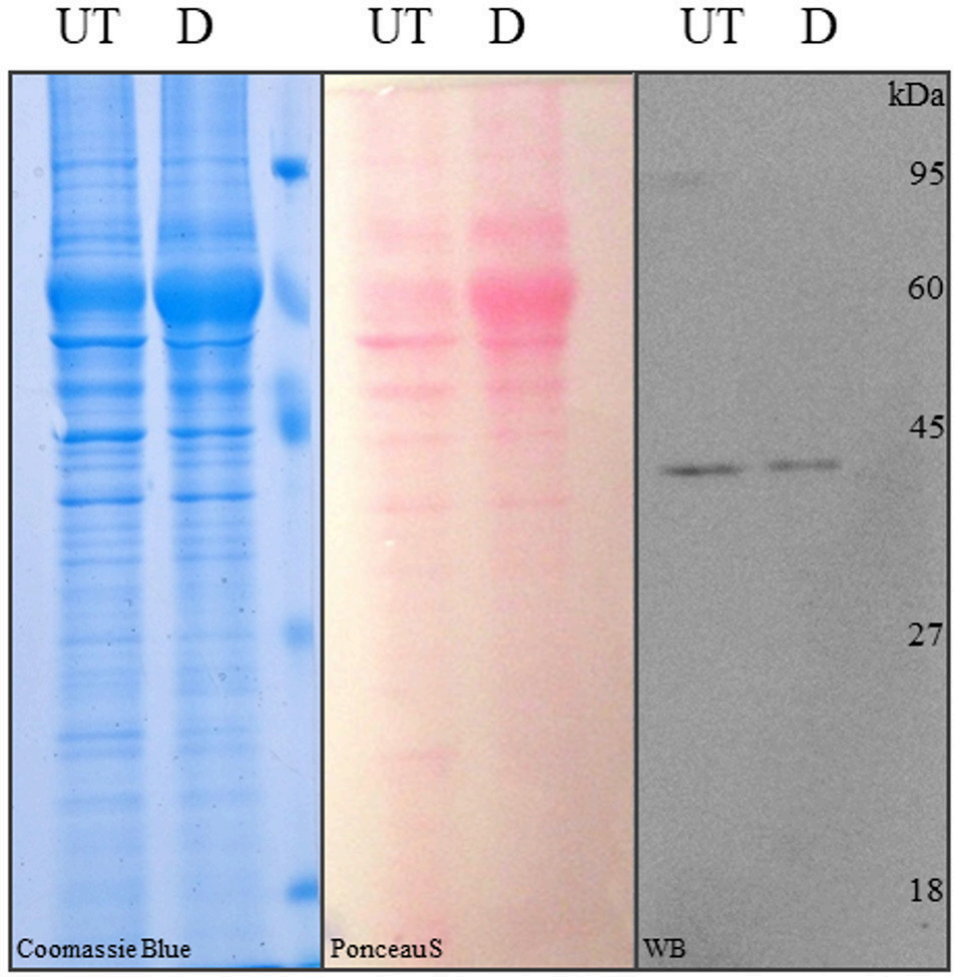
SDS-PAGE and Western blot analysis of differentiated and control hASCs. Proteins from whole cell lysates of untreated (UT) or differentiated (D) hASCs were solved in 12% acrylamide gels and stained with Coomasie blue (left image). The proteins were transferred to PVDF membranes from an unstained 12% acrylamide gel and stained with Ponceau S (central image). Immune anti-β-actin detection in hASCs whole cell lysates. 10 μg of whole cell lysate were loaded/lane. A representative image from three experiments is shown.

**Figure 6:**
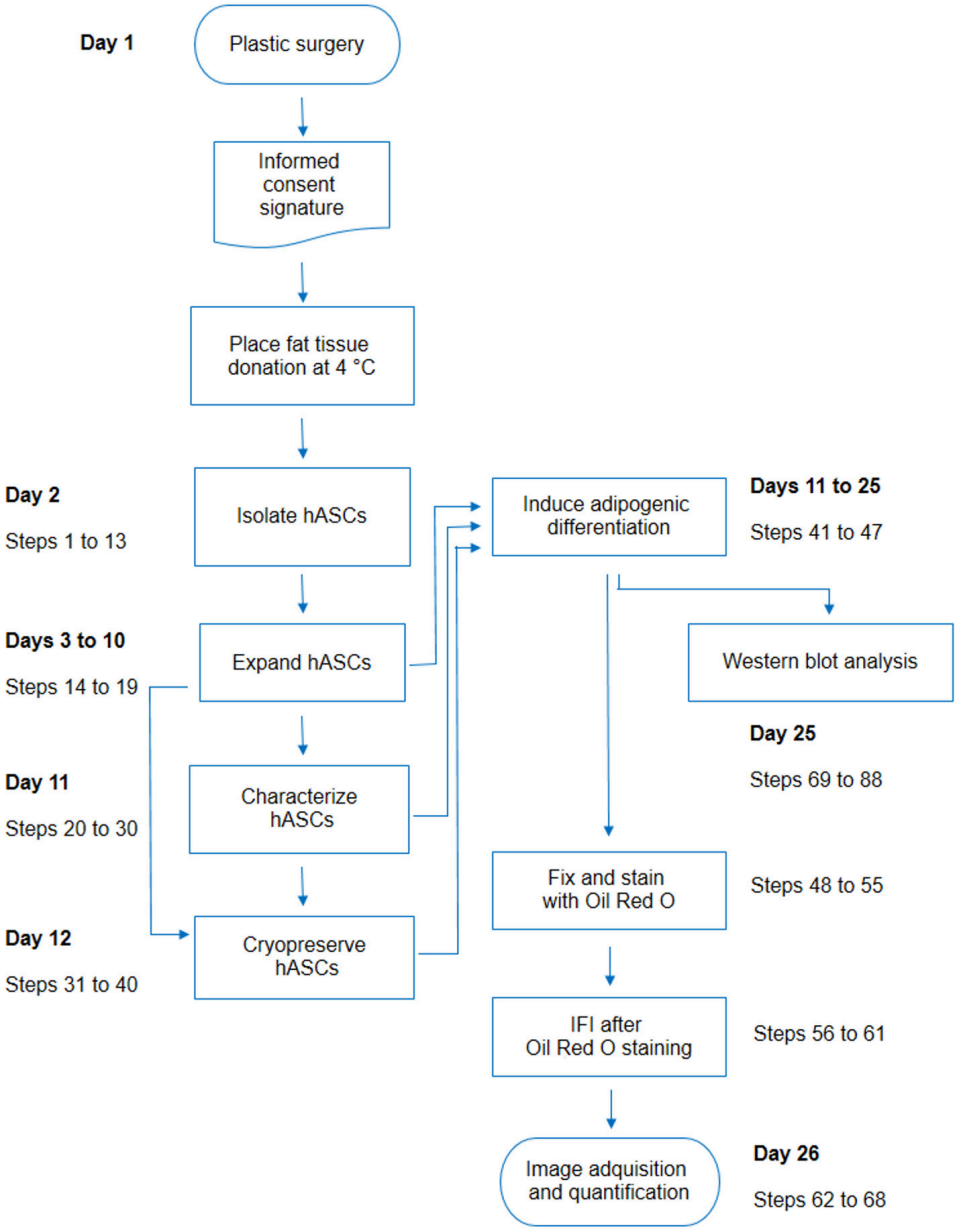
Flow chart including all steps described in this protocol. For each activity detailed in the protocol, the corresponding step numbers were indicated on the side, with an estimation of the earliest day after the plastic surgery in which the activity may be performed.

69. Cultivate the hASCs to 80% confluency in two 10 cm diameter culture plates. Cultivate one of them with 5% v/v FBS (untreated), and induce the other one to adipogenic differentiation following the same protocol explained above (steps 43–47). Cultivate under standard conditions (37°C and 5% CO_2_) until day 10.
70. Wash the monolayer gently one time with cold PBS buffer, and add 300 μL of lysis buffer (60 mM Tris-HCl, pH 6.8, 2% w/v SDS, 0.10 mM PMSF). Keep the cell culture plate on ice during this and the next step.
  ***ATTENTION*** SDS concentration in lysis buffer is critical to obtain a high quality protein extract in cells containing numerous and big size LDs, thus a high lipids/protein rate.
71. Detach the cells by using a cell scraper, homogenize by pipetting up and down, and transfer each lysate to a 1.5 mL microcentrifuge tube.
72. Heat the lysates for 10 min at 100°C, and then place them on ice.
73. Centrifuge at 13,000 × *g* for 10 min at 4°C. Take each supernatant and transfer them to new 1.5 mL conical microcentrifuge tubes.
  ***REST*** These samples may be stored at −20°C for weeks (or at −80°C for months, depending on the stability of the proteins of interest to analyze by WB, it may be stored for years).
74. Measure the total protein concentration by using a suitable method, as the Bicinchoninic acid assay (BCA assay). We employ the Pierce^TM^ BCA Protein Assay Kit (Thermo Fisher Scientific).
  ***ATTENTION*** Do not use the Bradford method for protein quantification of these lysates, because it is not compatible with the SDS concentration needed in the lysis buffer.
75. Prepare 12% SDS-PAGE Tris-Glycine gel, and assemble the electrophoresis chamber.
76. Prepare the samples by mixing the volume corresponding to 10 μg of each lysate with the appropriate volume of 5 X protein sample buffer (see RECIPE).
77. Heat the samples at 90–100°C for 5 min., load them on the gel and perform electrophoresis. We use initially 130 V, and when the samples reach the resolving gel, we increase to 190 V. With this settings, the electrophoresis takes 1 h 50 min.
78. Electro-transfer the proteins to a 0.22 μm PVDF (or nitrocellulose) membrane. Alternatively stain the gel with Coomassie G250 during 40 min., and then use discolor solution (5% v/v Acetic acid, 30% v/v ethanol) for 1 h until the background is pale and the protein bands are more visible.
  ***TIP*** In our system, PVDF membranes give better results than nitrocellulose, allowing us to load smaller amounts of proteins to detect by Western blot (i.e., we load 10 μg for PVDF and 40 μg for nitrocellulose membrane to get similar chemiluminescent signal).
79. If electro-transference is performed, use Towbin buffer (25 mM Tris-HCl, pH 8.30, 192 mM Glycine, 0.10% w/v SDS, 20% v/v methanol) and transfer ON at 10 V.
80. The next morning, take the membrane carefully out of the system and let it dry.
  ***REST*** Drying the membrane makes the proteins to bind stronger, and it is also an excellent storage method. The dried membrane may be stored at RT for weeks before blotting.
81. Incubate the membrane (only for PVDF membranes) in highly pure methanol for a few seconds to activate it. Briefly rinse it with water and stain with Ponceau S to evaluate the transfer quality. Remove excess of Ponceau S staining solution with water, take image and rinse off the Ponceau S stain with three PBS washes before starting the Western blot incubations.
82. Block the membrane by submerging it in 5% w/v non-fat dry milk in PBS at RT for 30 min.
83. Incubate with the primary antibody (anti-actin antibody, BD Biosciences, dilution 1:3,000) in PBS for 1 h.
84. Wash the membrane three times with PBST (0.01% v/v Tween-20 in PBS) for 5 min.
85. Incubate with an HRP-conjugated secondary antibody (anti-mouse IgG, Santa Cruz, dilution 1:2,500) in 5% w/v BSA/0.01% v/v PBST for 1 h.
86. Wash the membrane three times with PBST for 5 min, and a final wash with PBS.
87. Apply the chemiluminescent substrate and enhancer mixture (Super Signal® West Pico Chemiluminescent Substrate, Thermo Fisher Scientific) to the membrane and incubate 5 min.
88. Capture the chemiluminescent signal using a CCD camera-based equipment (ImageQuant™ LAS 4000—GE Healthcare Life Sciences) or similar, Figure 5.
  ***ATTENTION*** A flow chart resuming all steps described in this protocol can be checked in Figure 6.

## Anticipated results

hASCs are currently considered of high interest, because of their multipotency and bioactive molecules secretion capacities (Caplan and Correa, 2011). Figure 1 shows the isolation method of hASCs from human WAT described here, which yields ~1 × 10^4^ cells/g of tissue. Most articles cited the work of Zuk et al. (2001) as primary source, however, the procedure is not described in detail there. Our protocol will undoubtedly help filling this gap.

hASCs can be characterized by the expression of specific cell surface markers. We analyzed hASCs from 5 female individuals aged 25–65 years old. Figure 2 shows representative outputs of the flow-cytometry analysis performed for each cell population derived from a single patient. The results were either similar to those of Figure 2A or Figure 2B, differing mainly in the expression of CD105 marker. All were composed by more than 98% CD90+ (mean 98.38 ± 0.44%) and CD73+ (mean 98.74 ± 0.32%) cells, and <2% of cells containing markers of other cell types (negative cocktail, CD34, CD11b, CD19, CD45, and HLA-DR, average 1.48 ± 0.09%). For CD105, we obtained either one of two types of populations from each tissue sample: those with an average of 98.17 ± 0.49% of cells that express this marker (Figure 2A) and those with only 1.75 ± 0.35% of cells expressing it (Figure 2B). Bourin et al. (2013) suggested that it could be important distinguishing between the properties of CD105– and CD105+ hASCs populations. Indeed, murine ASCs (mASCs) CD105– were more prone to differentiate into adipocytes and osteocytes compared to CD105+ mASCs (Anderson et al., 2013). Whether this is the case or not in hASCs populations may be an interesting issue to analyze.

During adipogenic differentiation of hASCs, LDs increase in number and size. The adipogenic potential of hASCs tends to decline with consecutive passages under standard culture conditions (Li et al., 2017). To induce this process *in vitro*, we used low passage (up to 6) hASCs with an optimized drug cocktail (Gojanovich et al., 2016), based on a comparison of previously used formulations (Gimble and Guilak, 2003; Lee et al., 2004; Rodriguez et al., 2004; Dicker et al., 2005; Bunnell et al., 2008; Zhu et al., 2008; da Silva Meirelles and Covas, 2011; Kim et al., 2016). These include DMEM (high or low glucose), 10% v/v FBS, and variable concentrations of Insulin, Dexamethasone, Indomethacin, or Rosiglitazone, with or without 0.5 mM IBMX. We used the lowest concentration of each drug in a defined combination showing induction of LDs formation in a time lapse similar to previously reported (LDs becomes visible 5 days after induction). Also, the alternation of induction/maintenance medium (Figure 3) is distinctive to most previous methods, excepting the published by Qian et al. (2010), which uses induction cycles on hBMSCs. To prevent the cell detachment, hASCs where fixed and ORO-stained before reaching the mature adipocyte stage, which is characterized by a single LD and a peripheral nucleus. Although ORO-staining requires care to avoid dye precipitation, once this obstacle is overcome, this cost-effective stain is as useful as the newest and expensive lipid-dyes, with the additional advantage that it can be observed by both bright-field and fluorescence microscopy. Here, we show that ORO-staining does not interfere with the IF technique, and at the same time the later does not disturb the LD's integrity nor produce cells delipidization. Immediately after ORO-staining, the IF technique was applied on the same hASCs monolayers. Figure 4A (first pannel) shows actin disruption upon adipogenic differentiation (Yang et al., 2014). Additionally, to test our protocol for LDs (ORO) and cellular proteins (IF) double-staining, we selected antibodies against proteins from different subcellular locations (Figure 4A). Although purified LDs have been reported to recruit the Early Endosome Antigen 1 (EEA1) (Zehmer et al., 2009), we did not observe changes in EEA1 distribution after adipogenic differentiation (Figure 4A, second panel). Additionally, Figure 4A (third panel, inset) shows LDs immersed in the Golgi area (GM130 marker) in differentiated hASCs. Indeed, during LDs biogenesis in adipocytes, small nascent LDs are transported from the ER to the Golgi, where more TAGs are loaded and additional proteins are attached (Hesse et al., 2013). Later, LDs fusion and degradation are regulated by LD-coating proteins as Adipocyte differentiation-related protein (ADRP), among others (Hesse et al., 2013). This explains co-localization (yellow colored) signal by superposition of LDs (red) and ADRP (green) signals (Figure 4A, fourth panel, merge). Images also suggested the presence of nuclear LDs, which have been recently described (Layerenza et al., 2013). Figure 4B represents the quantification of ORO fluorescence in untreated and adipogenic differentiated hASCs.

Finally, we addressed a problem that although not mentioned in the literature, is well-known among researchers working with adipose differentiated hASCs. This is that lysates from those cells, prepared with typical RIPA lysis buffer (50 mM Tris-HCl, pH 7.5, 150 mM NaCl, 1% v/v NP40, 0.5% w/v Na Deoxicholate, 0.1% w/v SDS) or variants (i.e., with the addition of 0.1% v/v Triton X-100, or SDS increased to 1% w/v), are not well-resolved in acrilamide-bisacrilamide Tris-glycine or tris-tricine gels, resulting in the accumulation of proteins (including actin) in a single un-resolved band. Protein precipitation with 5% w/v TCA was partially useful, but a lot of protein was lost in the process. A much simpler solution was to increase the SDS concentration in the lysis buffer up to 2% w/v and boil the sample for 10 min before clarification. This procedure was the only one, allowing the proteins from adipogenic differentiated hASCs lysates to be efficiently resolved by SDS-PAGE. This can be observed in a CBB-stained gel (Figure 5, left panel), Pounceau S-stained PVDF membrane (Figure 5, central panel) and WB detection with actin-specific antibody (Figure 5, right panel).

In conclusion, our report deals with different important issues raised when working with hASCs, including details regarding isolation, characterization, adipogenic differentiation, ORO-IF combined double staining, and WB analysis (Figure 6). Given the growing interest in hASCs for research or clinical purposes, this report will positively impact on a broad public of researchers.

## Notes

### Troubleshooting

#### No adherent cells are found 24 h after the procedure

**Possible cause 1:** Inadequate enzyme concentration. Solution: Optimize enzymes concentrations. Especially collagenase lots may vary in activity, and too much activity may kill the cells. However, too low activity would not digest enough the tissue to for an adequate stem/stromal cells release.

**Possible cause 2:** Inadequate enzyme incubation time. Solution: Optimize enzymes incubation time. The incubation time may also depend on the enzyme lots and should be adjusted if necessary. Long time incubation may kill the cells and too short may not be enough for an adequate stem/stromal cell release.

**Possible cause 3:** The cellular pellet (SVF) has been lost. Solution: This pellet is labile; thus, extreme care should be taken when aspirating the media from above to avoid aspirating it.

**Possible cause 4:** The processing time took long time, which may kill the cells. Solution: Reduce the processing time. This will arrive with practice, or alternatively increase the ratio operator/tissue quantities. An experienced operator could efficiently process in average 100 g of adipose tissue, and this would take ~5 processing hours. If processing 200 g of adipose tissue, it should only be prolonged for 1 h, as only the mechanical processing steps will be significantly increased.

**Possible cause 5:** The sample was not properly prepared in the operating room. Solution: No antiseptic should be added (in some liposuction procedures the sample is mixed with an antiseptic solution). It is also common the addition of formol to fix the samples for anatomopathology analysis; be sure this is not done. The sample must be conserved without additives at room temperature or 4°C, and never be frozen for this procedure. The sample should be processed within no more than 3 days, this is especially critical in lipoaspirate samples, in which the cells are more exposed and unprotected as in the solid tissue niche.

**Possible cause 6:** Red blood cells have not been washed away, which may kill the hASCs. Solution: Make sure to eliminate most red blood cells by washing exhaustively the next day after the isolation procedure. These blood cells, may rapidly consume media nutrients, which may not be available for hASCs.

#### The culture gets contaminated

If this occurs on the first few days after the procedure, contamination may have become with the sample. Review with the nurse the way the samples are collected.

If contamination occurs later, make sure to use correct aseptic technique and test media, serum and other reagents to find the contamination source. Use antibiotics appropriately.

## Author contributions

AG, MG, LD, and MU: Conceived and designed the experiments; AG, MG, DM, and TR: Performed the experiments; AG, MG, LD, and MU: Analyzed the data; AG, LD, DB, and MU: Wrote the paper; RD, LD, DB, and MU: Supervised the study and corrected the manuscript.

### Conflict of interest statement

The authors declare that the research was conducted in the absence of any commercial or financial relationships that could be construed as a potential conflict of interest.
